# P100: Efficacy of alcohol-based and non-alcohol hand rubs after a single use and repeated use

**DOI:** 10.1186/2047-2994-2-S1-P100

**Published:** 2013-06-20

**Authors:** DR Macinga, S Edmonds, R McCormack

**Affiliations:** 1 GOJO Industries, Inc., Akron, OH, USA; 2 Northeast Ohio Medical University, Rootstown, OH, USA; 3 Bioscience Labs, Bozeman, OH, USA

## Introduction

Alcohol-based hand rubs (ABHR) vary in the type and concentration of alcohol used, and may include secondary active ingredients such as chlorhexidine gluconate (CHG). Alcohol-free handrubs based on quaternary ammonium compounds (QAC) may also be found in healthcare settings.

## Objectives

The objective of this study was to compare the efficacy of hand rubs containing different active ingredients after both single and repeated use at application volumes and test conditions closely representing clinical use conditions.

## Methods

Five commercial handrubs; Product A (70% ethanol), Product B (62% ethanol), Product C (63% 2-propanol), Product D (0.13% benzalkonium chloride (BAK)), and Product E (70% 2-propanol + 0.5% CHG) were evaluated according to ASTM E 2755. Fifteen test subjects evaluated each product at an application volume of 1.5 ml, which was rubbed over all surfaces of the hands until dry. Products were evaluated after a single application and after 10 hand contamination and product application cycles. All subjects used an internal reference (1.5 ml of 60% 2-propanol, rubbed until dry) prior to test product evaluation. Log_10_ reductions from baseline were calculated and statistical analysis conducted to compare products to each other and each product to its reference.

## Results

After a single use, ABHR obtained reductions equivalent to the internal reference. Product D (0.13% BAK) obtained a 1.70 log_10_ reduction, which was inferior to the reference and all ABHR (P<0.05). After 10 consecutive uses, log_10_ reductions for products A, B, C, D, and E were 4.37, 1.86, 3.82, 1.28, and 1.45, respectively. Products A and C were statistically superior to the reference, whereas Products B, D, and E were statistically inferior to the reference. Product A was statistically superior to all other test products.

## Conclusion

Alcohol-based handrubs performed similarly after a single use but significantly different with repeated use. Alcohol type and concentration were not strong predictors of product performance and inclusion of CHG did not improve efficacy over repeated use. These data indicate that overall product formulation is a critical component of product efficacy, and it is inappropriate to assume product performance based solely on the identity and concentration of active ingredients.

## Disclosure of interest

None declared

